# Prosthetic Joint Infection Complicated by Staphylococcus aureus Bacteremia and Tricuspid Valve Infective Endocarditis: A Novel Case Report

**DOI:** 10.7759/cureus.64821

**Published:** 2024-07-18

**Authors:** Hoore Jannat, Hamad Ahmad

**Affiliations:** 1 Internal Medicine, Khyber Medical College, Peshawar, PAK; 2 Internal Medicine, Westchester Medical Center, Valhalla, USA

**Keywords:** septic emboli, mssa bacteremia, prosthetic joint infection, total joint arthroplasty, tricuspid valve infective endocarditis

## Abstract

Prosthetic joint infection (PJI) is defined as an infection involving the prosthesis and surrounding soft tissue and bone that is a difficult complication to treat and is a common cause of revision total joint arthroplasty (TJA). Bacteremia, sepsis, and infective endocarditis (IE) are rare complications in patients who have undergone TJA. We report a rare case where a patient presented with purulent discharge from the left knee joint post-TJA concerning PJI and was found to have methicillin-sensitive *Staphylococcus aureus* bacteremia, tricuspid valve endocarditis, and septic pulmonary emboli. The patient underwent irrigation, debridement, and a spacer device placement in the affected knee joint for PJI and was medically treated for IE with six weeks of antibiotic therapy. The patient successfully recovered and was discharged to a rehabilitation facility. We conclude that PJI and IE secondary to TJA are very rare, but given the high morbidity and mortality, if diagnosis and treatment are delayed, physicians should always remain vigilant for these complications in the appropriate clinical context.

## Introduction

Prosthetic joint infection (PJI), also referred to as periprosthetic infection, is defined as an infection involving the joint prosthesis and adjacent tissue. In the United States, around 1.5 million total hip arthroplasties (THA) and total knee arthroplasties (TKA) are performed annually, with numbers increasing each year [1]. Prosthetic joint infection (PJI) is a difficult complication to treat and is a common cause of revision total joint arthroplasty (TJA). Around 2% of patients who undergo TJA develop PJI [2]. Obesity, diabetes mellitus, rheumatoid arthritis, exogenous immunosuppressive medications, and malignancy have been associated with an increased risk of PJI [2]. Common symptoms of PJI include joint pain, swelling, and drainage. Other potential complications include bacteremia, sepsis, and IE. The incidence of surgically acquired sepsis has decreased 10-fold to around 0.5% or less, but it still remains a significant potential complication [3]. Advanced surgical procedures, perioperative prophylactic antibiotic use, and enhanced sterile techniques contribute to the decrease in the incidence of sepsis and PJI [3]. Concurrent PJI and IE, though very rare, carry high mortality, with a one-year mortality for PJI reported at 3-8% and a six-month mortality for IE as high as 27% [4,5]. Prompt diagnosis and treatment are of key importance in such cases.

## Case presentation

**Figure 1:**
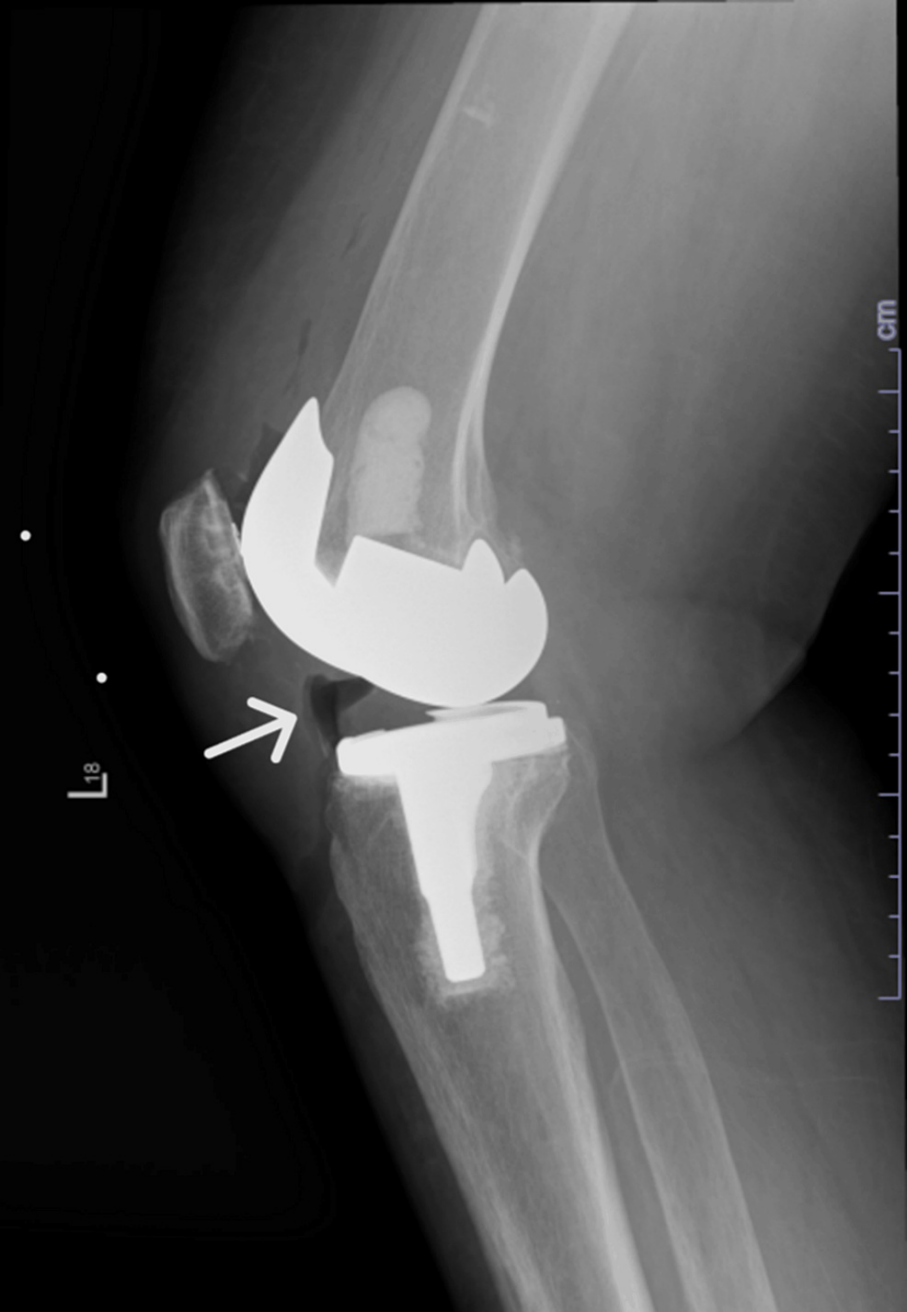
X-ray of left knee joint (lateral view) The white arrow is pointing to the development of gas within the joint.

We present a 69-year-old female with a history of breast cancer, peripheral vascular disease, hypertension, and left total knee arthroplasty who presented to the emergency department one month ago with wound dehiscence and purulent discharge from the left knee joint. On examination, the left knee was erythematous, swollen, and tender on palpation. The remainder of the systemic review and physical examination were unremarkable. Initial workup was significant for white blood cell count of 16x10^3^/mm^3^, C-reactive protein of 15 mg/dl, and erythrocyte sedimentation rate of 50 mm/h. Blood chemistry was essentially normal. X-rays of the left knee joint showed pneumarthrosis and fluid collection at the wound site concerning PJI (Figure 1).

**Figure 2:**
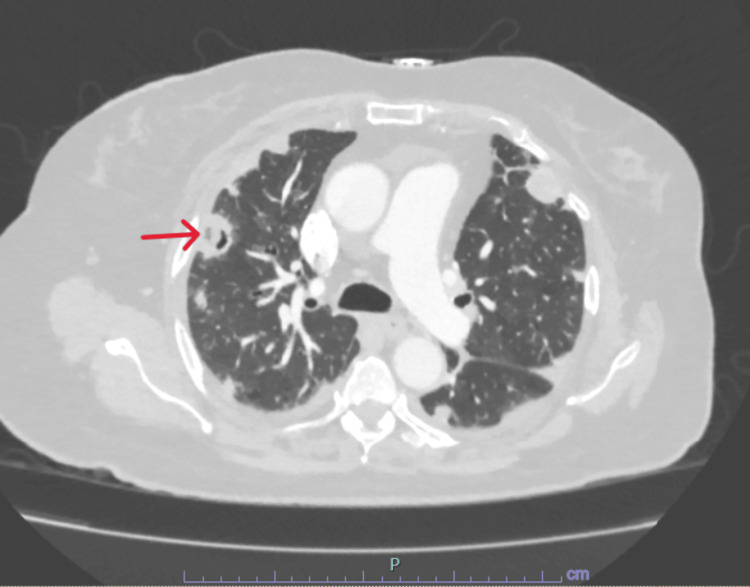
Computed tomography (CT) scan of the chest (image 1) The red arrow is pointing to a cavity lesion in the right upper lobe, likely septic emboli.

**Figure 3:**
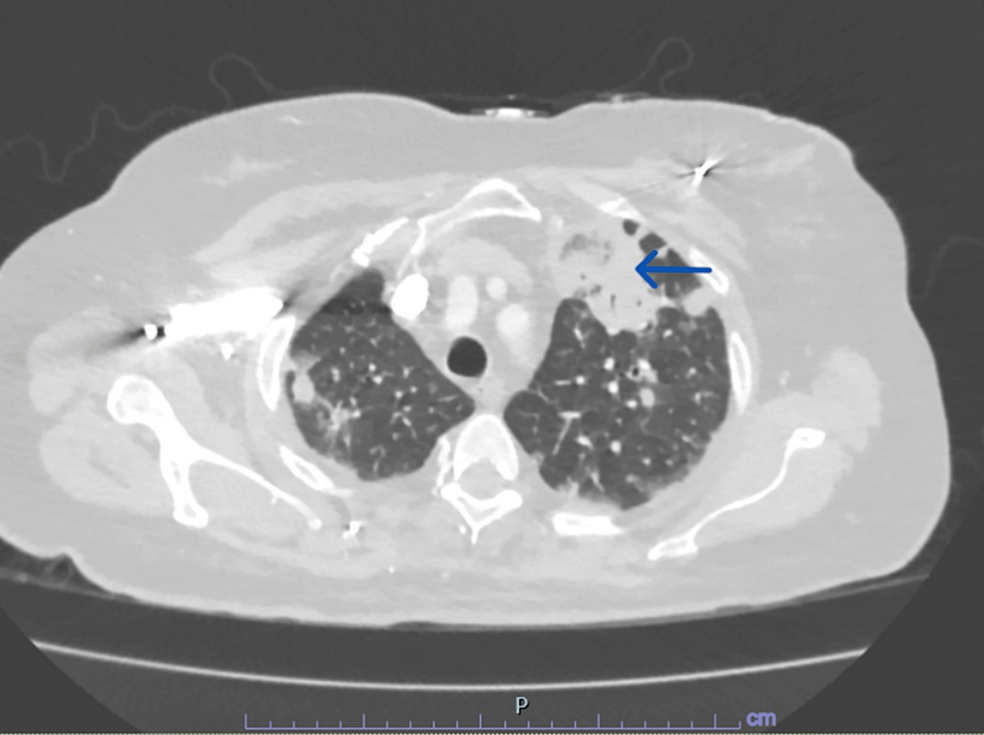
Computed tomography (CT) scan of the chest (image 2) The blue arrow is pointing to a large, hyperlucent lesion in the right upper lobe, likely septic emboli.

Given the concern for a possible prosthetic joint infection, infectious work up including blood cultures and wound cultures was obtained, and empiric intravenous broad-spectrum antibiotics were initiated. The next day, the patient underwent irrigation, debridement, and a spacer placement in the affected knee joint. Blood culture grew methicillin-sensitive Staph aureus, and antibiotics were tailored according to sensitivities. Computed tomographic (CT) scan of the thorax, abdomen, and pelvis for any other possible source of bacteremia and/or seeding showed multiple cavitary lung lesions highly suspicious for septic emboli (Figures 2, 3). Despite pulmonary involvement, respiratory status remained stable at baseline.

Given bacteremia and septic pulmonary emboli, transthoracic echocardiography was performed which showed tricuspid valvular vegetation consistent with infective endocarditis. Transesophageal echocardiography confirmed the presence of tricuspid vegetation without any valvular dehiscence or abscess formation. Mitral or aortic valves did not show any signs for endocarditis. Repeat blood cultures on antibiotics were consistently negative and patient remained clinically stable. No further purulence noted at the left knee joint with good wound healing. During hospitalization, patient was found to have a left lower extremity non-occlusive thrombus and was subsequently started on low-molecular-weight heparin. Patient was bridged to Warfarin and discharged to a subacute rehabilitation center on antibiotics for a total of six months of antibiotic therapy.

## Discussion

Prosthetic joint infection (PJI) is a serious complication of total joint arthroplasty (TJA) [6]. The risk of PJI is greater for knee arthroplasty than hip arthroplasty [7]. Risk factors include the presence of comorbidities such as rheumatoid arthritis, diabetes mellitus, malignancy, chronic kidney disease, the use of steroids, prior joint infections, prolonged duration of surgery, and bacteremia [8]. Our patient had a history of breast cancer in the past with no active disease, otherwise had no risk factors for PJI, and had no acute complications after joint arthroplasty. The incidence of concurrent infective endocarditis and prosthetic joint infection is not a very common entity, but it is associated with high morbidity and mortality, as presented by Humphrey et al. [9].

Isolated right-sided infective endocarditis, which includes tricuspid and pulmonic valves, accounts for approximately 10% of all cases of infective endocarditis [10]. Risk factors for tricuspid valve endocarditis include intravenous drug use, the presence of intracardiac electronic devices, and an underlying cardiac anomaly or bacteremia secondary to any other pathologic process such as osteomyelitis, pressure ulcers, or pyelonephritis [11]. Our patient did not have any of these risk factors but rather developed a PJI one month after TJA, which was further complicated by a bloodstream MSSA infection leading to tricuspid valve endocarditis. Few other cases of tricuspid valve endocarditis have been reported that resulted from infection elsewhere. Sami et al. reported a case of tricuspid valve IE in a non-IV drug user after a breast skin abscess [12]. Similarly, Hirakawa et al. reported another case of tricuspid valve IE in a patient with an abscess of an endogenous arteriovenous fistula in a chronic hemodialysis patient [13]. Very rarely, it can happen without any known risk factors, as was reported by Andrijašević et al. in a young, healthy patient without any significant medical history or risk factors [14].

The diagnosis of right-sided infective endocarditis is established based on clinical manifestations, blood cultures, and echocardiography as described in the Duke's criteria [15]. Our patient had positive blood cultures that grew MSSA and had echocardiographic evidence of endocardial involvement. Management of right-sided IE requires parenteral antibiotics, the removal of intravascular devices, and treating any possible sources of bacteremia, such as osteomyelitis, pneumonia, urinary tract infection, or surgical site infection [16]. Surgical intervention may be warranted in some cases with very large vegetation (>20 mm), recurrent septic pulmonary emboli, persistent bacteremia, or the presence of a highly resistant organism [16]. Our patient did not have any indication for surgical intervention for the IE, but she did undergo irrigation and debridement of the affected knee joint for PJI and source control. Our patient responded appropriately to treatment and improved.

## Conclusions

Our case report emphasizes the critical importance of promptly recognizing and managing periprosthetic joint infection (PJI) and its potential systemic complications. The development of bacteremia, endocarditis, and septic emboli secondary to PJI is rare but poses a life-threatening complication and can lead to significant morbidity and mortality. These complications need prompt diagnosis and treatment with a multidisciplinary approach. Additionally, further studies focusing on strategies to reduce these complications are essential to preventing such complications. This comprehensive approach is vital for optimizing patient outcomes and mitigating the risks associated with these serious complications.
